# The Costs and Cardiovascular Benefits in Patients With Peripheral Artery Disease From a Fourth-Generation Synchronous Telehealth Program: Retrospective Cohort Study

**DOI:** 10.2196/24346

**Published:** 2021-05-18

**Authors:** Jen-Kuang Lee, Chi-Sheng Hung, Ching-Chang Huang, Ying-Hsien Chen, Hui-Wen Wu, Pao-Yu Chuang, Jiun-Yu Yu, Yi-Lwun Ho

**Affiliations:** 1 Division of Cardiology Department of Internal Medicine National Taiwan University Hospital Taipei Taiwan; 2 Department of Laboratory Medicine National Taiwan University College of Medicine Taipei Taiwan; 3 Telehealth Center National Taiwan University Hospital Taipei Taiwan; 4 Cardiovascular Center National Taiwan University Hospital Taipei Taiwan; 5 Department of Internal Medicine College of Medicine National Taiwan University Taipei Taiwan; 6 Department of Nursing National Taiwan University Hospital Taipei Taiwan; 7 Department of Business Administration College of Management National Taiwan University Hospital Taipei Taiwan

**Keywords:** peripheral artery disease, fourth-generation synchronous telehealth program, ischemic stroke, hospitalization, cardiovascular disease, telehealth, stroke, cost, benefit, heart

## Abstract

**Background:**

Patients with peripheral artery disease (PAD) are at high risk for major cardiovascular events, including myocardial infarction, stroke, and hospitalization for heart failure. We have previously shown the clinical efficacy of a fourth-generation synchronous telehealth program for some patients, but the costs and cardiovascular benefits of the program for PAD patients remain unknown.

**Objective:**

The telehealth program is now widely used by higher-risk cardiovascular patients to prevent further cardiovascular events. This study investigated whether patients with PAD would also have better cardiovascular outcomes after participating in the fourth-generation synchronous telehealth program.

**Methods:**

This was a retrospective cohort study. We screened 5062 patients with cardiovascular diseases who were treated at National Taiwan University Hospital and then enrolled 391 patients with a diagnosis of PAD. Of these patients, 162 took part in the telehealth program, while 229 did not and thus served as control patients. Inverse probability of treatment weighting (IPTW) based on the propensity score was used to mitigate possible selection bias. Follow-up outcomes included heart failure hospitalization, acute coronary syndrome, stroke, and all-cause readmission during the 1-year follow-up period and through the last follow-up.

**Results:**

The mean follow-up duration was 3.1 (SD 1.8) years for the patients who participated in the telehealth program and 3.2 (SD 1.8) for the control group. The telehealth program patients exhibited lower risk of ischemic stroke than did the control group in the first year after IPTW (0.9% vs 3.5%; hazard ratio [HR] 0.24; 95% CI 0.07-0.80). The 1-year composite endpoint of vascular accident, including acute coronary syndrome and stroke, was also significantly lower in the telehealth program group after IPTW (2.4% vs 5.2%; HR 0.46; 95% CI 0.21-0.997). At the end of the follow-up, the telehealth program group continued to exhibit a significantly lower rate of ischemic stroke than did the control group after IPTW (0.9% vs 3.5%; HR 0.52, 95% CI 0.28-0.93). Furthermore, the medical costs of the telehealth program patients were not higher than those of the control group, whether in terms of outpatient, emergency department, hospitalization, or total costs.

**Conclusions:**

The PAD patients who participated in the fourth-generation synchronous telehealth program exhibited lower risk of ischemic stroke events over both mid- and long-term follow-up periods. However, larger-scale and prospective randomized clinical trials are needed to confirm our findings.

## Introduction

Cardiovascular (CV) diseases remain the biggest health burden worldwide [1]. Telemedicine can be used to monitor diseases and treat patients in real time. Previous studies have shown that patients with CV diseases who received telehealth medicine had better control over vascular risk factors, such as hypertension, diabetes mellitus, and dyslipidemia [2]. Furthermore, both Spyros et al [3] and Sally et al [4] reported that telemedicine was an important prognostic factor for patients with congestive heart failure in reducing all-cause mortality Relatedly, we have shown that a fourth-generation telehealth program—more specifically, an internet-based, synchronized disease management program that provides an immediate response—is associated with lower all-cause mortality among patients with CV as compared to that seen in patients who did not participate in the program [5]. However, Takahashi et al [6] found that asynchronous telemonitoring did not lead to fewer hospitalizations or emergency department visits. Meanwhile, a review article suggested that telemedicine should be carefully evaluated and applied in only those patients who would really benefit from it in terms of improved clinical outcomes [7].

Patients with peripheral artery disease (PAD) have relatively high rates of various comorbidities, including acute coronary syndrome (ACS), stroke, and congestive heart failure [8-10]. Numerous cohort studies have reported that the clinical outcomes of PAD patients are grave, with the rates of major adverse CV events and mortality being higher among PAD patients than among the general population. Moreover, PAD is an independent risk factor for mortality in patients with CV diseases [11]. Current guidelines advocate the use of antiplatelet agents and statins as primary therapies for these patients to prevent further major adverse cardiovascular events, because they are at high risk of CV events [11,12]. Therefore, it is important to treat PAD patients using innovative modalities to improve their overall prognosis. New treatment strategies that offer better CV outcomes for these patients are needed in the internet era. The costs and CV benefits of the fourth-generation synchronous telehealth program for these patients remain unknown.

The aims of this study were to elucidate the effects of the fourth-generation synchronous telehealth program for PAD patients on their CV outcomes, including stroke, ACS, and hospitalization for heart failure; and to evaluate the medical costs for these patients, including those who are treated at outpatient clinics and emergency departments or who are hospitalized.

## Methods

### Study Design

This was a retrospective cohort study in which data from a tertiary hospital in Taiwan was analyzed. This study was approved by the Institutional Review Board of National Taiwan University Hospital (NTUH), Taipei, Taiwan. All protocols were performed in accordance with relevant guidelines and regulations.

### Patient Selection

Patients older than 20 years diagnosed with chronic CV diseases were screened at NTUH. Chronic CV diseases included coronary artery disease, myocardial infarction, heart failure, PAD, stroke, and hypertension. The patients with PAD were further selected for this study, with the PAD patients who participated in the fourth-generation synchronous telehealth program at the telehealth center of NTUH being enrolled as the study group. The patients with PAD who received usual care were enrolled as the control group. The decision of whether to participate in the telehealth program depended on the patients and/or their caregivers.

### Telehealth Care Program

The fourth-generation synchronous telehealth program at our center is an integrated remote management program for chronic diseases. The internet-based platform was developed by the Graduate Institute of Biomedical Electronics and Bioinformatics, National Taiwan University, Taiwan. The details of this program have been reported previously [13]. Briefly, this telehealth program provides the following services: the collection of biometric data including single-lead electrocardiography, blood pressure, heart rate, and oximetry data, which are transferred from patients to our telehealth center daily and on demand; daily and on-demand telephone calls from nurse case managers for communication and health promotion; care and guidance from full-time nurse case managers and cardiologists who are in charge of care 24 hours per day; and long-term medication services and care management, which are discussed with the patients’ primary care physician after acute events. The telehealth program bridges acute and home care and emphasizes education, prevention, and the early detection of clinical deterioration.

### Usual Care

The patients in the control group received the usual care provided by the primary care physicians at our CV center according to updated guidelines including, but not limited to, the American Heart Association’s guidelines for lifestyle modification and primary prevention to reduce CV risks, guidelines for the management of stable ischemic heart disease, and the American Diabetes Association’s guidelines for the management of diabetes. These patients made routine outpatient department visits (once every 3 months) to their primary care physicians. There was no contact between the telehealth center and the patients receiving usual care.

### Covariates and Outcomes

The covariates were demographics (age, sex, and BMI), 10 comorbidities (hypertension, diabetes mellitus, hyperlipidemia, coronary artery disease, chronic kidney disease, myocardial infarction, stroke, heart failure, atrial fibrillation, and cancer), Charlson Comorbidity Index score, vital signs and laboratory data (mean arterial pressure, pulse rate, creatinine, and low-density lipoprotein-cholesterol), and the kinds of medication used at the time of enrollment. The outcomes were all-cause readmission, heart failure hospitalization, ACS, stroke, and the composite of ACS and stroke during follow-up. Each outcome was defined according to whether the patient was admitted with a principal discharge diagnosis during hospitalization.

### Statistical Analysis

To reduce possible potential confounding when comparing the risk of outcomes between the telehealth and control groups, we employed an inverse probability of treatment weighting (IPTW) based on the propensity score [14]. The propensity score was estimated using a multivariable logistic regression model without consideration to interaction effects among the covariates, with the study group (1=telehealth, 0=control) being regressed on the selected covariates listed in Table 1 and the follow-up duration being replaced with the index date. We used the weight to estimate the average treatment effect and used a stabilized weight to mitigate the impact of extreme estimated propensity scores [15]. The balance of covariates between the groups before and after IPTW was checked using the absolute value of standardized difference between the groups, where a value less than 0.1 was considered a negligible difference [16].

The incidence of outcomes during follow-up was calculated using the incidence density ID expressed as number of events per 100 person-years. The absolute risk difference between the groups was also calculated in the IPTW-adjusted cohort. Furthermore, we compared the risk of outcomes during follow-up between the groups using the Cox proportional hazards model. The outcomes were assessed during the 1-year follow-up period, during the entire follow-up period, and before and after the 1-year follow-up period (landmark by 1 year). The average number of all-cause readmissions during follow-up between groups was compared between the groups using a Poisson model which treated the logarithm of follow-up duration as an offset variable. The accumulated medical expenditures during follow-up was compared between the groups using a linear regression model. The study group was the only explanatory factor in the aforementioned regression models. A 2-sided *P* value <.05 was considered statistically significant, and no adjustment of multiple testing (multiplicity) was made. All statistical analyses were performed using SAS version 9.4 (SAS Institute).

**Table 1:** Baseline characteristics of the study patients with peripheral arterial occlusive disease who participated (n=162) and did not participate (n=229) in the telemedicine program.

Variable		Before IPTW^a^				After IPTW		
		With^b^ (n=162)	Without^c^ (n=229)	STD^d^	With (n=162)		Without (n=229)	STD
Age (years), mean (SD)		70.8 (12.5)	70.0 (12.7)	0.06	70.1 (12.4)		70.2 (12.5)	–0.01
Male sex, n (%)		103 (63.6)	141 (61.6)	0.04	62.3		63.0	–0.01
Body mass index (kg/m^2^), mean (SD^d^; n=374)		24.7 (4.4)	24.5 (4.0)	0.06	24.9 (4.6)		24.6 (3.9)	0.07
**Comorbid conditions, n (%)**								
	Hypertension	107 (66.0)	148 (64.6)	0.03	66.1		64.9	0.03
	Diabetes mellitus	68 (42.0)	100 (43.7)	–0.03	44.2		43.6	0.01
	Hyperlipidemia	76 (46.9)	100 (43.7)	0.07	43.2		44.0	–0.02
	Coronary artery disease	108 (66.7)	145 (63.3)	0.07	65.8		65.6	<0.01
	Chronic kidney disease	49 (30.2)	60 (26.2)	0.09	27.1		28.9	–0.04
	Old myocardial infarction	17 (10.5)	27 (11.8)	–0.04	10.6		11.9	–0.04
	Stroke	53 (32.7)	65 (28.4)	0.09	29.9		29.8	<0.01
	Heart failure	38 (23.5)	57 (24.9)	–0.03	22.4		24.1	–0.04
	Atrial fibrillation	17 (10.5)	29 (12.7)	–0.07	14.5		11.6	0.09
	Cancer	13 (8.0)	22 (9.6)	–0.06	8.7		9.0	–0.01
Charlson Comorbidity Index score, mean (SD)		3.1 (2.0)	3.0 (2.1)	0.05	3.0 (2.0)		3.1 (2.1)	–0.04
**Laboratory data, mean (SD)**								
	Mean arterial pressure, (mmHg; n=354)	90.0 (14.2)	89.6 (14.3)	0.02	90.6 (13.6)		89.7 (14.1)	0.07
	Pulse, (mmHg; n=354)	74.6 (12.9)	75.9 (14.0)	–0.10	75.8 (13.6)		75.8 (13.8)	<0.01
	Creatinine, (mg/dL; n=385)	2.1 (2.8)	2.3 (2.6)	–0.07	2.1 (2.7)		2.4 (2.6)	–0.09
	LDL–C^e^ (mg/dL; n=318)	90.3 (28.2)	88.2 (28.8)	0.07	91.9 (27.4)		88.8 (29.1)	0.11
**Medication, n (%)**								
	ACEI^f^/ARB^g^	82 (50.6)	121 (52.8)	–0.04	53.9		52.4	0.03
	Beta blocker	96 (59.3)	129 (56.3)	0.06	60.7		58.5	0.05
	dCCB^h^	87 (53.7)	115 (50.2)	0.07	51.3		52.4	–0.02
	Diuretics	73 (45.1)	95 (41.5)	0.07	43.3		43.2	<0.01
	Aspirin	81 (50.0)	125 (54.6)	–0.09	52.6		53.9	–0.02
	Clopidogrel/Cilostazol	73 (45.1)	130 (56.8)	–0.24	51.3		51.9	–0.01
	Oral anticoagulants	25 (15.4)	33 (14.4)	0.03	13.3		13.5	–0.01
	Oral hypoglycemic agent	44 (27.2)	63 (27.5)	–0.01	27.1		27.9	–0.02
	Insulin	12 (7.4)	29 (12.7)	–0.18	10.7		10.5	<0.01
	Statin	70 (43.2)	97 (42.4)	0.02	39.3		41.9	–0.05
	Follow-up period (years), mean (SD)	3.1 (1.8)	3.2 (1.8)	–0.09	3.3 (1.9)		3.1 (1.7)	0.14

^a^IPTW, inverse probability treatment weighting.

^b^Treated with the telemedicine program.

^c^Treated without the telemedicine program.

^d^STD: standardized difference.

^e^LDL-C: low-density lipoprotein-cholesterol.

^f^ACEi: angiotensin converting enzyme inhibitor.

^g^ARB: angiotensin receptor blocker.

^h^dCCB: dihydropyridine calcium channel blocker.

## Results

### Patient Demographics and Clinical Features

**Figure 1:**
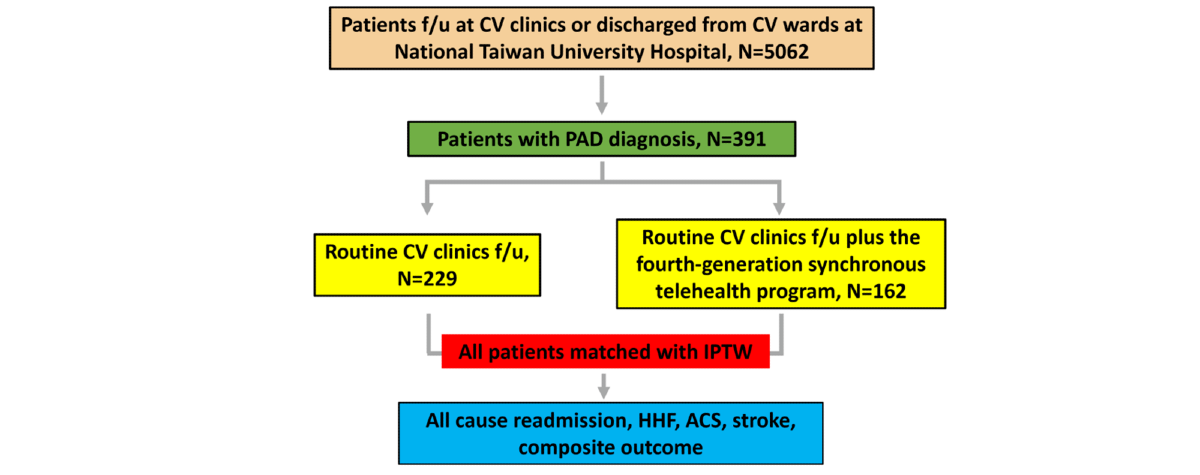
Flow chart of enrolled patients. ACS: acute coronary syndrome; CV: cardiovascular: f/u: follow-up; HHF: hospitalization for heart failure; IPTW: inverse probability of treatment weighting; PAD: peripheral artery disease.

A total of 5062 patients were screened in this study, and 391 patients with PAD diagnosis were further selected into the telehealth and control group (Figure 1). The baseline characteristics of those patients are reported in Table 1. In the telehealth groups, the mean age was 70.8 (SD 12.5) years, and 63.6% (103/162) of the patients were male. In the control group, the mean age was 70.0 (SD 12.7) years, and 61.6% (141/229) of the patients were male. The telehealth group had more patients with stroke (53/162, 32.7% vs 65/229, 28.4%, in the telehealth group vs control group, respectively), chronic kidney disease (49/162, 30.2% vs 60/229, 26.2%), coronary artery disease (108/162, 66.7% v. 145/229, 63.3%), and hyperlipidemia (76/162, 46.9% vs 100/229, 43.7%). The Charlson Comorbidity Index score was higher in the telehealth group (mean 3.1, SD 2.0) than in the control group (mean 3.0, SD 2.1). The mean follow-up time was 3.1 (SD 1.8) years for the telehealth group and 3.2 (SD 1.8) years for the control group. After propensity score weighting, the 2 study groups were found to have well-balanced characteristics (Table 1).

### Prognosis of Patients With PAD Participating in the Telehealth Program

During the 1-year follow-up period, 2 of the 162 (1.2%) patients in telehealth group and 8 of the 229 (3.5%) patients in the control group had stroke, respectively. The 1-year stroke rate was significantly lower in the telehealth group (0.9 vs 3.5 events per 100 person-years; hazard ratio [HR] 0.24; 95% CI 0.07-0.80) after IPTW. The incidence of composite vascular outcome in the telehealth cohort was also lower than that in the control cohort after IPTW (2.7 vs 5.8 events per 100 person-years; HR 0.46; 95% CI 0.21-0.997). However, no significant differences between the 2 were observed in terms of all-cause readmission, heart failure hospitalization, or ACS in the 1-year follow-up period. At the end of the follow-up period, only the stroke rate was significantly lower in the telehealth group (1.4 vs 2.6 events per 1000 person-years; HR 0.52; 95% CI 0.28-0.93) after IPTW (Table 2 and Multimedia Appendix 1).

**Table 2:** Follow-up outcomes (after inverse probability treatment weighting) in patients with peripheral arterial occlusive disease who participated (n=162) and did not participate (n=229) in the telemedicine program.

Outcome		Telemedicine event rate, %/ID^a^ (95% CI)	Nontelemedicine, event rate, %/ID (95% CI)	ARD^b^ (95% CI)	HR^c^ of telemedicine (95% CI)	*P* value
**One-year follow-up**						
	All-cause readmission	37.8/55.8 (46.8 to 64.8)	39.3/59.6 (50.2 to 69.0)	–0.38 (–1.68 to 0.92)	0.93 (0.75 to 1.17)	.55
	HFH^d^	3.7/4.1 (2.3 to 6.8)	4.3/4.8 (2.8 to 7.7)	–0.71 (–3.84 to2.42)	0.85 (0.42 to 1.73)	.66
	ACS^e^	1.6/1.7 (0.6 to 3.7)	2.3/2.5 (1.1 to 4.7)	–0.76 (–2.88 to 1.36)	0.69 (0.25 to 1.94)	.48
	Stroke	0.9/0.9 (0.2 to 2.6)	3.5/3.9 (2.1 to 6.6)	–2.99 (–5.29 to 0.68)	0.24 (0.07 to 0.80)	.02
	Composite outcome	2.4/ 2.7 (1.3 to 5.0)	5.2/ 5.8 (3.6 to 8.9)	–3.14 (–6.19 to 0.09)	0.46 (0.21 to 0.997)	.049
**End of follow-up**						
	All-cause readmission	62.2/ 35.5 (31.2 to 40.3)	62.6/ 38.1 (33.4 to 43.1)	–2.5 (–9.1 to –4.0)	0.95 (0.79 to 1.13)	.56
	HFH	10.1/ 3.1 (2.2 to 4.3)	7.6/ 2.6 (1.7 to 3.7)	0.55 (–0.79 to1.90)	1.25 (0.78 to 2.02)	.36
	ACS	3.0/ 0.9 (0.5 to 1.6)	2.9/ 1.0 (0.5 to 1.7)	–0.04 (–0.80 to 0.73)	0.96 (0.43 to 2.17)	.92
	Stroke	4.4/ 1.4 (0.8 to 2.2)	7.7/ 2.6 (1.8 to 3.7)	–1.24 (–2.37 to 0.11)	0.52 (0.28 to 0.93)	.03
	Composite outcome	7.5/ 2.3 (1.6 to 3.4)	9.4/ 3.2 (2.3 to 4.4)	–0.86 (–2.20 to 0.48)	0.72 (0.44 to 1.17)	.18

^a^ID: incidence density.

^b^ARD: absolute risk difference.

^c^HR: hazard ratio.

^d^HHF: hospitalization for heart failure.

^e^ACS: acute coronary syndrome.

### Landmark Analysis by 1-Year Follow-Up

**Figure 2:**
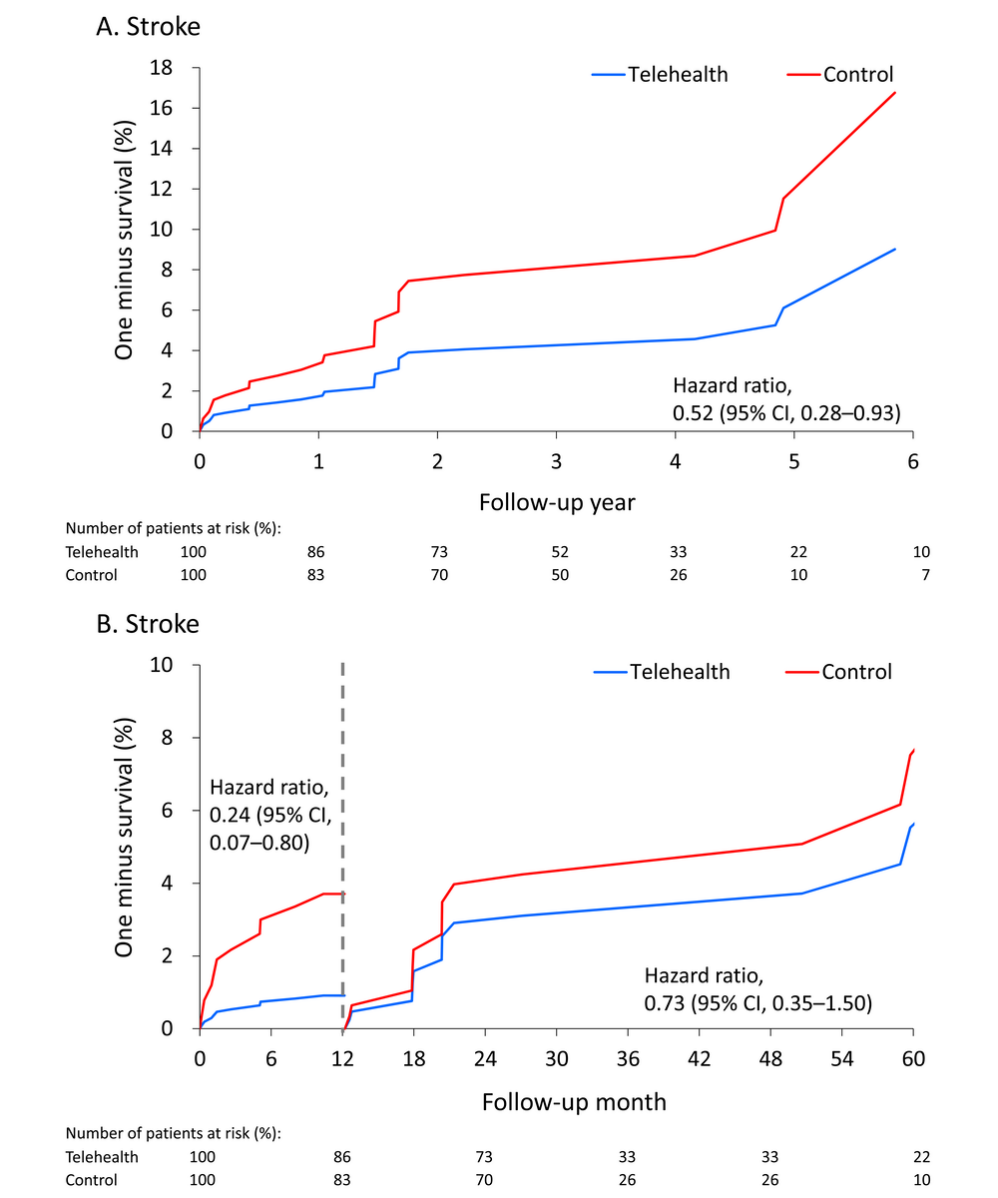
Fitted (predicted) survival curves of stroke during the overall follow-up (A) and stratified by 1-year follow-up (B) in patients who participated in the telemedicine program and who did not participate the telemedicine program.

**Figure 3:**
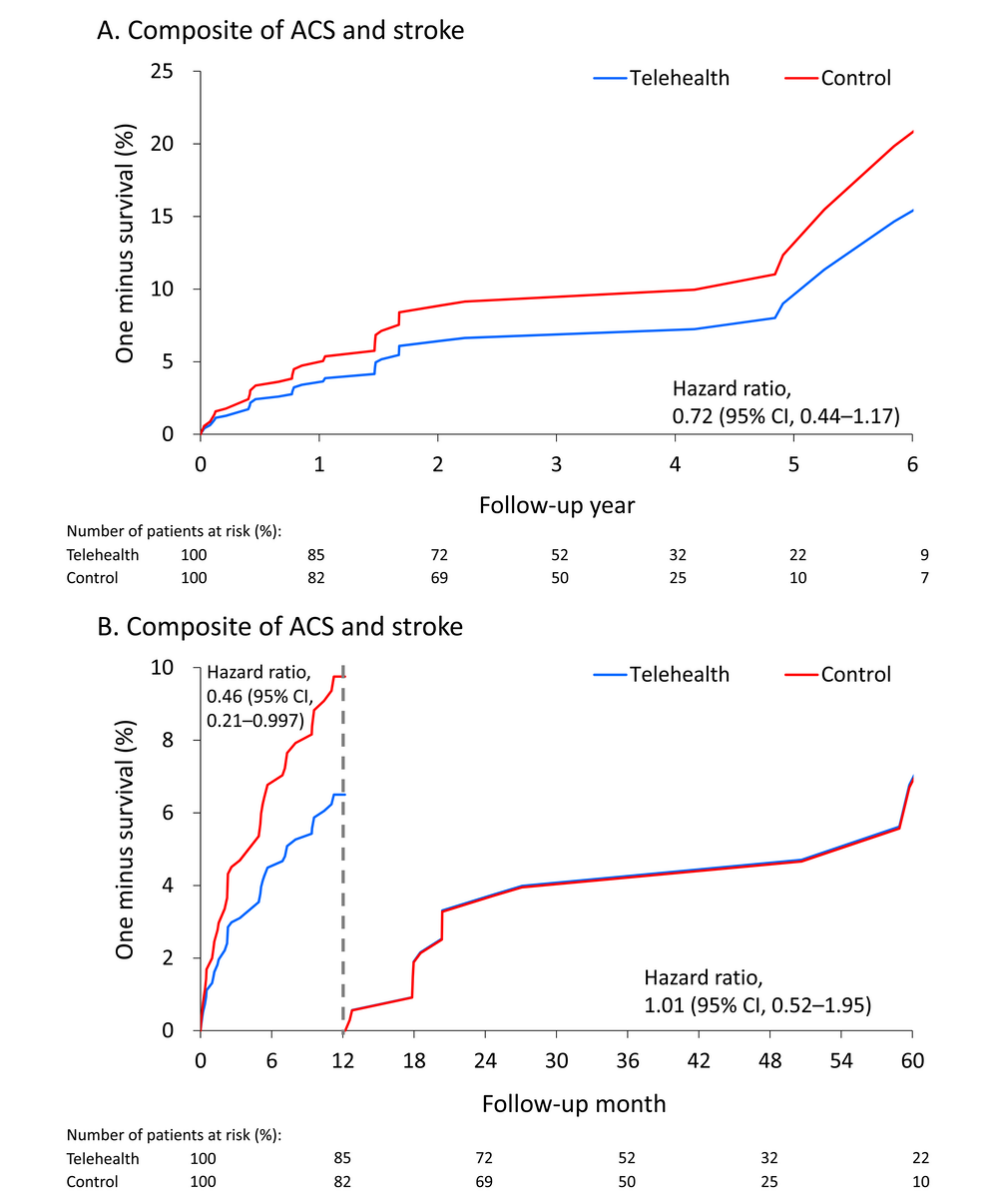
Fitted (predicted) survival curves of composite arterial cardiovascular outcome during the overall follow-up (A) and stratified by 1-year follow-up (B) in patients who participated in the telemedicine program and who did not participate the telemedicine program.

The fitted (predicted) survival curve of stroke during the overall follow-up period was significantly lower in the telehealth group than in the control group (Figure 2A). The 2 curves remained significantly different during the 1-year follow-up period (HR 0.24; 95% CI 0.07-0.80) but were not significantly different afterwards (HR 0.73; 95% CI 0.35-1.50; Figure 2B). Similarly, the fitted (predicted) survival curve for composite vascular outcome (including acute coronary syndrome and stroke) during the overall follow-up period was not significantly lower in the telehealth group than in the control group (Figure 3A). The 2 curves were only significantly different in the 1-year follow-up after being stratified by 1-year follow-up (HR 0.46; 95% CI 0.21-0.997; Figure 3B).

### Medical Utilization and Costs for Patients Participating in the Telehealth Program

The PAD patients in both the telehealth group and the control group had similar readmission times (mean 1.6, SD 2.0 vs mean 1.7, SD 2.2; rate ratio 1.08; 95% CI 0.97-1.20). Furthermore, no significant differences in medical expenditures between the 2 groups were observed in terms of outpatient, emergency department, hospitalization, or total costs during the whole course of follow-up (Table 3 and Multimedia Appendix 1).

**Table 3:** Medical utilization and cost (after inverse probability treatment weighting) in patients with peripheral arterial occlusive disease who participated (n=162) and did not participate (n=229) in the telemedicine program after inverse probability treatment weighting..

Outcome		Telemedicine, mean (SD)	Nontelemedicine, mean (SD)	RR^a^ or *B*^b^ of telemedicine (95% CI)	*P* value
Number of readmissions		1.8 (2.3)	1.6 (2.1)	1.08 (0.97 to 1.20)	.16
**Medical expenditures (NTD^c^×10^3^)**					
	Outpatient	577 (959)	528 (891)	577 (963)	.66
	Emergency department	42 (55)	46 (63)	42 (54)	.32
	Hospitalization	760 (853)	913 (1236)	819 (914)	.57
	Total	1380 (1486)	1487 (1692)	1438 (1495)	.88

^a^RR: rate ratio.

^b^*B*: regression coefficient.

^c^NTD: New Taiwan dollar.

### Incidence Rate of All Outcomes in Patients Who Participated in the Telehealth Program

**Figure 4:**
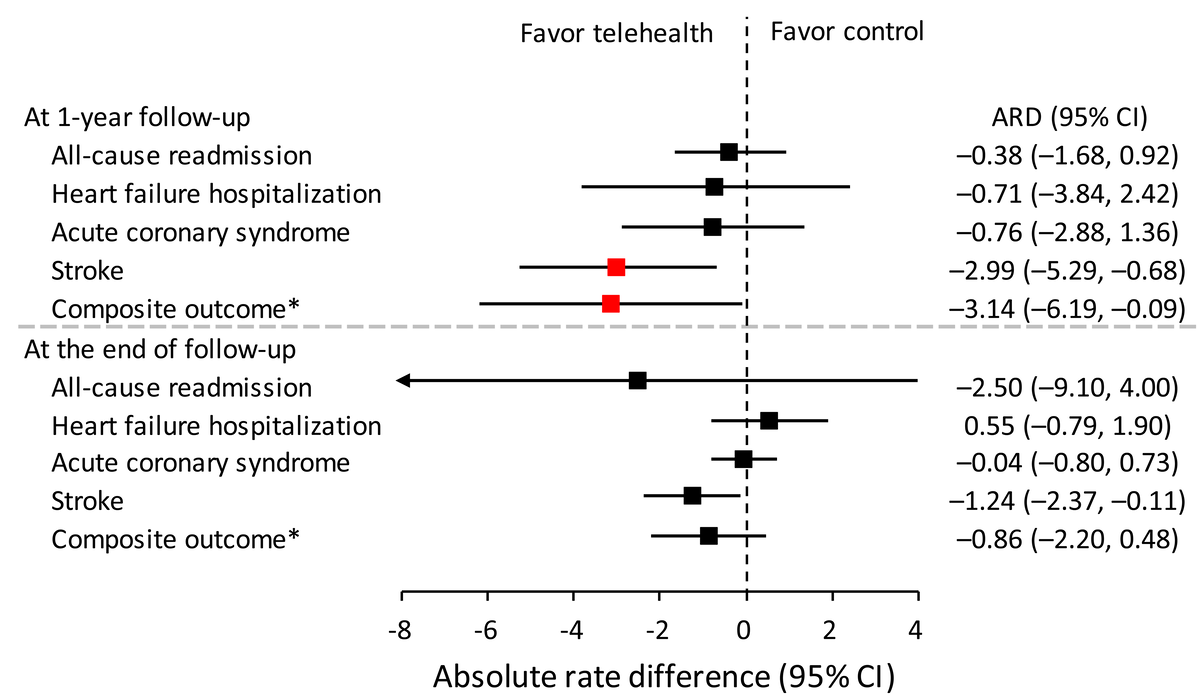
The difference of the incidence rate (expressed as number of event per 100 person-years) of all outcomes in patients who participated in the telemedicine program and who did not participate in the inverse probability of treatment weighting–adjusted cohort. ACS: acute coronary syndrome; ARD: absolute risk difference. *Composite of ACS and stroke.

The absolute risk difference in the incidence (expressed as number of events per 100 person-years) of stroke and arterial vascular composite outcome was –2.99 (95% CI –5.29 to –0.68) and –3.14 (95% CI –6.19 to –0.09), respectively, at the 1-year follow-up. At the end of the follow-up period, only the absolute risk difference of the incidence rate for stroke was significant (Figure 4).

## Discussion

This is the first study to investigate the application of the fourth-generation synchronous telehealth program to patients with PAD in terms of various CV outcomes. Retrospectively, we found that the PAD patients who participated in the telehealth program had lower risks of both stroke and composite vascular events in the midterm follow-up but only a lower risk of stroke events over the long-term follow-up period. The vascular protection seemed most prominent in the first year, and this phenomenon then diminished in the following years with regular CV clinic visits. In the first year, 3 out of every 100 patients in the telemedicine group were free from stroke events. Otherwise, the total medical costs were comparable in both groups.

Telehealth care has been shown to reduce hospitalizations in patients with chronic conditions such as asthma, chronic obstructive pulmonary disease, and heart failure [17-20]. We have previously reported that better cost-effectiveness and clinical outcomes were noted with the use of fourth-generation synchronous telehealth program in patients with chronic CV diseases [13]. However, some other studies failed to show better clinical outcomes in patients taking part in telehealth programs [6,7]. This difference in the results could be explained by the enrollment of different types of patients and the implementation of different versions of telehealth programs. We previously reported that PAD was the remaining prognostic factor for CV admission in patients receiving telehealth care after multivariable Cox regression [21]. However, the costs and CV benefits of the fourth-generation synchronous telehealth program for PAD patients are still not well validated. In this study, we focused on those patients with PAD who had higher risks of grave CV outcomes such as myocardial infarction and stroke [22,23]. According to a previous study, the 5-year cumulative incidence of mortality among asymptomatic and symptomatic patients with PAD is 19% and 27%, respectively [24]. In the internet era, it is important to consider the speed of medical care responses of telehealth programs, especially with respect to high-risk patient groups. The early identification of PAD patients with optimized and comprehensive treatment is critical for improving clinical outcomes, and telehealth programs could be applied to PAD-related wound care or gangrene management in place of CV systems that provide comprehensive care [25,26]. In this study, we found that patients receiving telemedicine care had significantly lower rates of stroke events in the mid- and long-term follow-up periods than did the group that received normal care. As high blood pressure is a major cause of stroke, better control of blood pressure is essential to preventing stroke attacks. Lu et al [27] showed that the implementation of home telehealth care supervised by nursing managers improved blood pressure control in patients with high blood pressure. Furthermore, it was found that a mobile phone intervention could significantly reduce systolic blood pressure among patients with high blood pressure (mean difference of 4.3 mmHg) [28]. Digital health strategies were also found to have a beneficial effect on blood pressure control [29]. These findings suggest that the stroke prevention benefits of telemedicine may be linked to the better blood pressure control exhibited by patients using the telehealth program. On the other hand, the prevalence of carotid artery stenosis is high in patients with PAD [30]. Through good medication compliance, the early management of CV risk factors, and the prompt detection of clinical arrhythmia, full-time nursing case managers and cardiologists who provide round-the-clock care are also able to reduce the risk of stroke among those patients with PAD that have carotid artery stenosis.

In this study, we found that, in the midterm, the fourth-generation synchronous telehealth programs improved the composite vascular outcome (including acute coronary syndrome and stroke) of patients with PAD. This vascular protection was most prominent in the first year and gradually diminished over the years that followed as confirmed through the regular follow-up visits at CV clinics. The telehealth program seems to bridge the transition from hospital admission to home care. Therefore, the proper timing for initiating telehealth is just as important as the timing for taking medication. The conditions of patients with CV disease deteriorated rapidly just after their discharge [31,32]. Our study supports the notion that the optimal timing for implementing a telehealth program for patients with PAD is at the time of discharge from hospital.

Those patients with PAD who took part in the telehealth program in this study had 2.99 fewer stroke events and 1.86 fewer composite vascular events per 100 patients in the first year than did the control group patients. As the prevalence and incidence of patients with PAD is high, the number of patients needed to be treated with telehealth program can be easily achieved in clinical practice. As patients participating in telehealth programs will have to pay additional fees, the costs and benefits associated with this program should be analyzed. We found that the medical costs of the telehealth program patients in this study were not greater than those of the control group, in spite of the fact that they did have better clinical outcomes in terms of stroke and composite vascular events. This finding suggests that the participation of PAD patients in the telehealth programs can also confer economic benefits. Moreover, opportunity costs other than medical ones should also be considered. Because patients with PAD suffer from grave CV outcomes, they are more likely to lose their jobs and to pay higher insurance premiums. In the United States, individuals diagnosed with PAD face higher health care–related expenditures and out-of-pocket expenses compared to healthy adults [33]. Therefore, the total expenditure of patients with PAD and stroke is likely higher than that revealed in our study.

This study had several limitations. First, the study was not randomized, which resulted in the heterogeneity of the patient population and disease severity. However, we performed IPTW matching of 2 groups with balance to minimize the possible confounding effect of clinical factors. Second, the outcome of amputation was not coded in our cohort. In addition, although we did show the composite vascular outcome in our analysis, this outcome is particularly important in patients with PAD and should be clarified in a future study. Third, the clinical outcomes were derived from the electronic billing and medical records of our hospital, and the data for patients who received care outside of our hospital were not recorded. Resources that were used but not billed might have also been overlooked when extracting data from our billing system.

Those patients with PAD who participated in the fourth-generation telehealth program exhibited a lower risk of stroke events over both short- and long-term follow-up periods than did the control group. In addition, the total medical costs of the 2 groups were comparable, with no significant difference being found. Further randomized trials are needed, however, to confirm our findings and guide clinical practice.

## Appendix

Multimedia Appendix 1 Follow-up outcomes in patients with peripheral arterial occlusive disease who participated and did not participate in the telemedicine program; and medical utilization and cost in patients with peripheral arterial occlusive disease who participated and did not participate in the telemedicine program.

## Abbreviations

ACS: acute coronary syndrome
CV: cardiovascular
HR: hazard ratio
IPTW: inverse probability of treatment weighting
NTUH: National Taiwan University Hospital
PAD: peripheral artery disease
