# Supplementary material for: The Costs and Cardiovascular Benefits in Patients With Peripheral Artery Disease From a Fourth-Generation Synchronous Telehealth Program: Retrospective Cohort Study
Source: J Med Internet Res. 2021 May 18;23(5):e24346. doi: 10.2196/24346 (PMC8170551; doi:10.2196/24346)
Supplement: Multimedia Appendix 1 [file jmir_v23i5e24346_app1.docx]

**Table 2. Follow-up outcomes in patients with peripheral arterial occlusive disease who participated and did not participate in the telemedicine program**

| Outcome | | Data before IPTW^a^, n (%) | | Data after IPTW | | | | |
| --- | --- | --- | --- | --- | --- | --- | --- | --- |
|  | | Telemedicine  (n=162) | Nontelemedicine  (n=229) | Telemedicine  (n=162), event rate (%)/ID^b^ (95% CI)§ | Nontelemedicine (n=229), event rate (%)/ID (95% CI)§ | ARD^c^ (95% CI) | HR^d^ of telemedicine  (95% CI) | *P* value |
|  | |  |  |  |  |  |  |  |
| **One-year follow-up** | | | | | | | | |
|  | All-cause readmission | 56 (34.6) | 89 (38.9) | 37.8/55.8 (46.8 to 64.8) | 39.3/59.6 (50.2 to 69.0) | –0.38 (–1.68 to 0.92) | 0.93 (0.75 to 1.17) | .55 |
|  | HFH^e^ | 6 (3.7) | 11 (4.8) | 3.7/4.1 (2.3 to 6.8) | 4.3/4.8 (2.8 to 7.7) | –0.71 (–3.84 to2.42) | 0.85 (0.42 to 1.73) | .66 |
|  | ACS^f^ | 2 (1.2) | 5 (2.2) | 1.6/1.7 (0.6 to 3.7) | 2.3/2.5 (1.1 to 4.7) | –0.76 (–2.88 to 1.36) | 0.69 (0.25 to 1.94) | .48 |
|  | Stroke | 2 (1.2) | 8 (3.5) | 0.9/0.9 (0.2 to 2.6) | 3.5/3.9 (2.1 to 6.6) | –2.99 (–5.29 to 0.68) | 0.24 (0.07 to 0.80) | .020 |
|  | Composite outcome* | 4 (2.5) | 12 (5.2) | 2.4/ 2.7 (1.3 to 5.0) | 5.2/ 5.8 (3.6 to 8.9) | –3.14 (–6.19 to 0.09) | 0.46 (0.21 to 0.997) | .049 |
| **End of follow-up** | | | | | | | | |
|  | All-cause readmission | 94 (58.0) | 144 (62.9) | 62.2/ 35.5 (31.2 to 40.3) | 62.6/ 38.1 (33.4 to 43.1) | –2.5 (–9.1 to –4.0) | 0.95 (0.79 to 1.13) | .56 |
|  | HFH | 16 (9.9) | 18 (7.9) | 10.1/ 3.1 (2.2 to 4.3) | 7.6/ 2.6 (1.7 to 3.7) | 0.55 (–0.79 to1.90) | 1.25 (0.78 to 2.02) | .36 |
|  | ACS | 4 (2.5) | 7 (3.1) | 3.0/ 0.9 (0.5 to 1.6) | 2.9/ 1.0 (0.5 to 1.7) | –0.04 (–0.80 to 0.73) | 0.96 (0.43 to 2.17) | .92 |
|  | Stroke | 7 (4.3) | 17 (7.4) | 4.4/ 1.4 (0.8 to 2.2) | 7.7/ 2.6 (1.8 to 3.7) | –1.24 (–2.37 to 0.11) | 0.52 (0.28 to 0.93) | .03 |
|  | Composite outcome* | 11 (6.8) | 22 (9.6) | 7.5/ 2.3 (1.6 to 3.4) | 9.4/ 3.2 (2.3 to 4.4) | –0.86 (–2.20 to 0.48) | 0.72 (0.44 to 1.17) | .18 |

^a^ IPTW: inverse probability treatment weighting.

^b^ID: incidence density

^c^ARD: absolute risk difference

^d^HR: hazard ratio

^e^HHF: hospitalization for heart failure

^f^acute coronary syndrome

**Table 3.** Medical utilization and cost in patients with peripheral arterial occlusive disease who participate and did not participate in the telemedicine program.

| Outcome | | Data before IPTW^a^ | | Data after IPTW | | | |
| --- | --- | --- | --- | --- | --- | --- | --- |
|  | | Telemedicine  (n=162), mean (SD) | Nontelemedicine  (n=229), mean (SD) | Telemedicine, (n=162), mean (SD) | Nontelemedicine, (n=229), mean (SD) | RR^b^ or *B*^c^ of telemedicine  (95% CI) | *P* value |
|  | |  |  |  |  |  |  |
|  | Number of readmissions | 1.6 (2.0) | 1.7 (2.2) | 1.8 (2.3) | 1.6 (2.1) | 1.08 (0.97 to 1.20) | .16 |
| **Medical expenditures (NTD**^d^**×10^3^)** | | | | | | | |
|  | Outpatient | 577 (959) | 528 (891) | 577 (963) | 535 (903) | 42.0 (–143.8 to 227.9) | .66 |
|  | Emergency department | 42 (55) | 46 (63) | 42 (54) | 48 (65) | –6.0 (–17.8 to 5.9) | .32 |
|  | Hospitalization | 760 (853) | 913 (1236) | 819 (914) | 879 (1161) | –60.4 (–268.5 to 147.7) | .57 |
|  | Total | 1380 (1486) | 1487 (1692) | 1438 (1495) | 1462 (1651) | –24.3 –337.9 to 289.2) | .88 |

^a^IPTW: inverse probability treatment weighting.

^b^RR: rate ratio.

^c^*B*: regression coefficient.

^d^NTD: New Taiwan dollar.
